# Efficient Feeder-Free Episomal Reprogramming with Small Molecules

**DOI:** 10.1371/journal.pone.0017557

**Published:** 2011-03-01

**Authors:** Junying Yu, Kevin Fongching Chau, Maxim A. Vodyanik, Jinlan Jiang, Yong Jiang

**Affiliations:** Advanced Development Programs, Cellular Dynamics International, Inc., Madison, Wisconsin, United States of America; University of Southern California, United States of America

## Abstract

Genetic reprogramming of human somatic cells to induced pluripotent stem cells (iPSCs) could offer replenishable cell sources for transplantation therapies. To fulfill their promises, human iPSCs will ideally be free of exogenous DNA (footprint-free), and be derived and cultured in chemically defined media free of feeder cells. Currently, methods are available to enable efficient derivation of footprint-free human iPSCs. However, each of these methods has its limitations. We have previously derived footprint-free human iPSCs by employing episomal vectors for transgene delivery, but the process was inefficient and required feeder cells. Here, we have greatly improved the episomal reprogramming efficiency using a cocktail containing MEK inhibitor PD0325901, GSK3β inhibitor CHIR99021, TGF-β/Activin/Nodal receptor inhibitor A-83-01, ROCK inhibitor HA-100 and human leukemia inhibitory factor. Moreover, we have successfully established a feeder-free reprogramming condition using chemically defined medium with bFGF and N2B27 supplements and chemically defined human ESC medium mTeSR1 for the derivation of footprint-free human iPSCs. These improvements enabled the routine derivation of footprint-free human iPSCs from skin fibroblasts, adipose tissue-derived cells and cord blood cells. This technology will likely be valuable for the production of clinical-grade human iPSCs.

## Introduction

Human induced pluripotent stem cells (iPSCs), similar to human embryonic stem cells (ESCs), are capable of unlimited proliferation and have the potential to differentiate into all cell types of the body [1]–[2]. These cells, thus, have applications in basic biology, disease modeling, drug development, and transplantation therapies. By expressing a defined set of reprogramming factors, iPSCs have been generated from many cell types of different species [1]–[8]. Initial methods for iPSC generation employed genome-integrating retroviral or lentiviral vectors [2]–[3]. These approaches could produce tumorigenic insertional mutations, and residual or reactivation of transgene expression during iPSC differentiation could affect lineage choice and the functionality of iPSC derivatives [2], [9]. To overcome these problems, various methods were developed to derive iPSCs free of exogenous DNA (footprint-free), including repeated treatments with reprogramming factors (plasmids, minicircle DNA, non-integrating adenoviral vectors and proteins), transposons and RNA viral vectors [10]–[16]. However, these methods suffer one or more of the following limitations: the unacceptable low reprogramming efficiency; the labor-intensive removal of reprogramming factors from iPSCs; the requirement for viral packaging or feeder cells. Thus, there is a need to develop a simple and efficient feeder-free method to enable the routine derivation of footprint-free iPSCs from many human donor samples, and eventually the derivation of clinical-grade human iPSCs.

A recent report described the efficient derivation of footprint-free human iPSCs from fibroblasts using synthetic modified mRNA [17]. Compared to viral and DNA-based reprogramming methods, the mRNA-mediated transgene delivery offers a safer approach for the derivation of clinical-grade human iPSCs. The requirement for repeated transfections, however, limits the application of this method to cells types that are easily transfectable such as skin fibroblasts. It remains to be seen whether this method can be readily adapted to cells that are relatively resistant to lipid-mediated transfections, such as cells of hematopoietic lineages. In addition to the mutations arising during reprogramming, somatic mutations present in the donor cells may also significantly affect the safety of human iPSCs. Therefore, the selection of appropriate donor cell types will likely be important for the derivation of clinical-grade human iPSCs. A reprogramming method that is applicable to different cell types will be highly desirable to address this question. Additionally, recent data suggest the retention of donor cell epigenetic memory in early passage iPSCs [18], which influences their *in vitro* differential capacity. It remains to be seen whether this is affected by the specific methods employed in the derivation of iPSCs. Thus alternative methods are needed for the efficient derivation of human footprint-free iPSCs.

We have previously generated footprint-free human iPSCs using oriP/EBNA-1 (Epstein-Barr nuclear antigen-1) episomal vectors to deliver reprogramming genes (*OCT4*, *SOX2*, *NANOG*, *LIN28*, *c-MYC*, *KLF4* and *SV40LT*) [19]. Compared to other methods, this approach has several advantages. First, the oriP/EBNA-1 vectors have a wide host cell range, enabling the application of this method to many human cell types. Second, it does not require viral packaging. Third, no repeated treatments with reprogramming factors are needed. A single transfection of episomal vectors is sufficient for the derivation of human iPSCs. Moreover, higher transfection efficiency can be achieved with these vectors due to the oriP/EBNA-1-mediated nuclear import and retention of vector DNA [20]. Fourth, the oriP/EBNA-1 vectors replicate once per cell cycle and are generally present at low copy number per cell, thus minimizing DNA rearrangement and genome integration [21]. Last, the removal of episomal vectors from human iPSCs can be accomplished by simple cell culture without any additional manipulation, due to the silencing of the viral promoter driving EBNA-1 expression in iPSCs, and the inherent instability of oriP/EBNA-1 episomal state - stably established episomes are lost from cells at a rate of ∼5% per cell generation due to defects in vector synthesis and partitioning [22]. However, despite these advantages, our original episomal method yielded low reprogramming efficiency (∼3 iPSC colonies from ∼1×10^6^ input human foreskin fibroblasts), and used mouse embryonic fibroblast (MEF) feeder cells, which seriously limit the industrial and therapeutic applications of this method.

In this report, we have made significant improvement of the episomal reprogramming method. Using chemically defined media, we have established a small molecule-aided feeder-free reprogramming condition for the efficient derivation of footprint-free human iPSCs from skin fibroblasts, adipose tissue derived cells and cord blood cells. This method can be readily adapted to the derivation of clinical-grade human iPSCs. Of particular interest, iPSC derivation with this method appeared to progress through a distinct intermediate stage. It will be interesting to find out how this small-molecule aided episomal reprogramming method compares to other reprogramming methods in terms of the quality of iPSCs they generate.

## Results

### Identifying small molecules for improved episomal reprogramming

**Figure 1:**
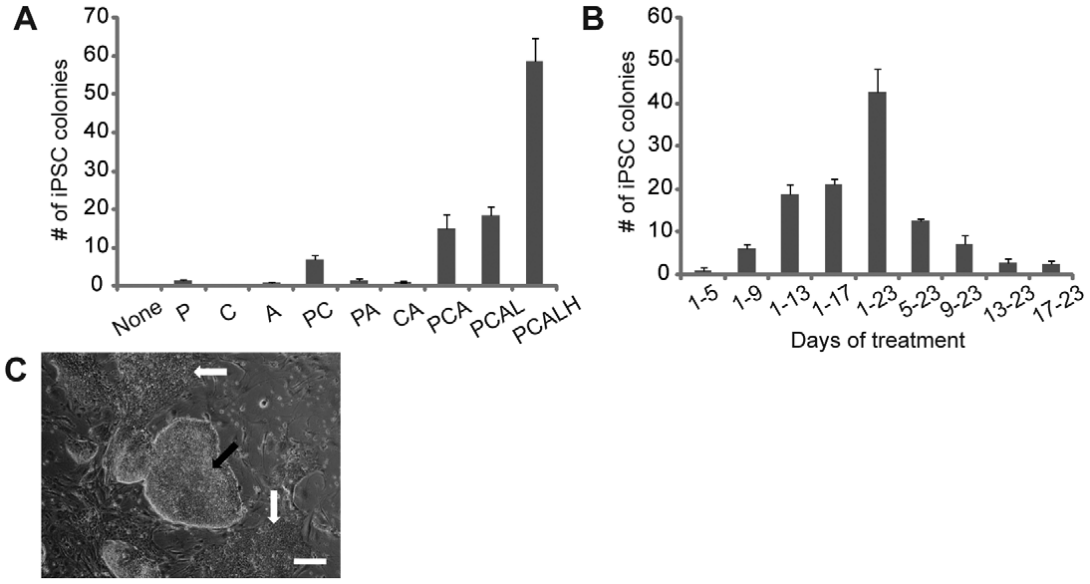
Improving episomal reprogramming efficiency with small molecules. (**A**) Effects of PD0325901 (P, 0.5 µM), CHIR99021(C, 3 µM), A-83-01(A, 0.5 µM), hLIF (L, 1000 U/ml) and HA-100 (H, 10 µM) on episomal reprogramming. Transfected human foreskin fibroblasts were plated to MEF feeder cells. MEF-conditioned human ESC medium supplemented with 100 ng/ml bFGF (CM100) was used to support reprogramming. Different combinations of small molecules and hLIF were added throughout reprogramming starting on day 4 post-transfection. (**B**) Temporal requirement of small molecule treatment for improved episomal reprogramming. PCALH was added to CM100 for different days during reprogramming. Alkaline phosphatase positive iPSC colonies with typical human ESC morphology (e.g. compact colonies, high nucleus-to-cytoplasm ratios and prominent nucleoli) were counted on day 22–23 post-transfection. The number of iPSC colonies was from ∼0.33×10^6^ input cells. Data shown are mean ± standard error (SEM) (n = 3). (**C**) Extensive differentiation of the newly derived iPSCs (p3) in the presence of small molecules. When expanded in human ESC medium or MEF-conditioned human ESC medium supplemented with small molecules on MEF feeder cells, the iPSCs derived with the continuous presence of small molecules exhibited extensive differentiation. The addition of bFGF in the culture medium had no effect. Black arrow: undifferentiated iPSC colonies; white arrows: differentiated colonies. Scale bars: 100 µm.

We have previously developed an oriP/EBNA-1-based episomal reprogramming approach for the derivation of footprint-free human iPSCs [19]. The reprogramming efficiency with this method, however, was prohibitively low (∼3 iPSC colonies from ∼1×10^6^ input fibroblasts). This low reprogramming efficiency was likely due to the loss of oriP/EBNA-1 vectors (>25% per cell generation) and DNA methylation-mediated transgene silencing during the first two weeks post-transfection, a time window required for successful iPSC generation [23]. Thus, small molecules that could either accelerate reprogramming process, or reduce episomal vector loss and transgene silencing during the first two weeks post-transfection, were expected to improve episomal reprogramming. To identify such small molecules, we tested chemical compounds that were previously implicated in reprogramming and epigenetic modifications, using a two-vector episomal combination containing expression cassettes for all seven transgenes (*OCT4*, *SOX2*, *NANOG*, *LIN28*, *c-MYC*, *KLF4* and *SV40LT*) (7F-1, Fig. S1A), human foreskin fibroblasts as donor cells and traditional reprogramming conditions (MEF feeder cells and human ESC medium). We found that the episomal reprogramming efficiency could be greatly enhanced with the addition of a MEK inhibitor PD0325901, a GSK3β inhibitor CHIR99021, and a TGF-β/Activin/Nodal receptor inhibitor A-83-01 (Fig. 1A). Previous studies showed that TGF-β signaling inhibitors together with the MEK inhibitor PD0325901 resulted in >100-fold increase in the viral reprogramming efficiency [24]. As shown in Figure 1A, the TGF-β signaling inhibitor A-83-01, either alone or together with the MEK inhibitor PD0325901, had minimal effect on episomal reprogramming. All three inhibitors PD0325901, CHIR99021, and A-83-01 were required to achieve the maximal increase in reprogramming efficiency. Human leukemia inhibitory factor (hLIF), though did not significantly improve episomal reprogramming efficiency, increased the proliferation of reprogramming intermediates and the reprogramming pace. The ROCK inhibitor HA-100, though had minimal effect on its own, further increased the episomal reprogramming efficiency in the presence of PD0325901, CHIR99021, A-83-01 and hLIF. The increase in the episomal reprogramming efficiency correlated with the duration of small molecule treatment (Fig. 1B). Early treatment, i.e. between day 1 and 5 post-transfection, though not sufficient on its own, appeared to be important for the maximal effect of small molecules on episomal reprogramming.

The observation that episomal reprogramming could be significantly improved by the continuous presence of PD0325901, CHIR99021, A-83-01 and hLIF raised an interesting question about the identity of iPSCs generated. Based on the gene expression and culture requirements, recent studies suggest that human ESCs are in a pluripotent state similar to mouse epiblast-derived stem cells (EpiSCs), and different from mouse ESCs derived from earlier blastocyst stage, which were proposed to be in a naïve ground state of pluripotency [25]. In the presence of PD0325901, CHIR99021, A-83-01 and hLIF, human ESCs and human ESC-like iPSCs differentiated rapidly. On the contrary, these molecules were able to expand mouse ESC-like human iPSCs from reprogramming cultures that were not previously exposed to these small molecules [26]. These mouse ESC-like human iPSCs readily differentiated following withdrawal of the small molecules. Surprisingly, the human episomal iPSCs obtained in the continuous presence of small molecules expanded well under conditions for human ESCs (without small molecules), but underwent extensive differentiation when cultured in the same condition used for their derivation, i.e. in the presence of small molecules (Fig. 1C), suggesting that these iPSCs were in a pluripotent state similar to human ESCs, not mouse ESCs. The seemingly conflicting results could be explained by the presence of activities that mitigated the effectiveness of small molecules in the MEF feeder cells and human ESC medium used for reprogramming (e.g. ligands for TGF-β signaling), which might enable the generation of human ESC-like pluripotent state in the presence of small molecules.

### Developing a feeder-free episomal reprogramming condition with defined media

**Figure 2:**
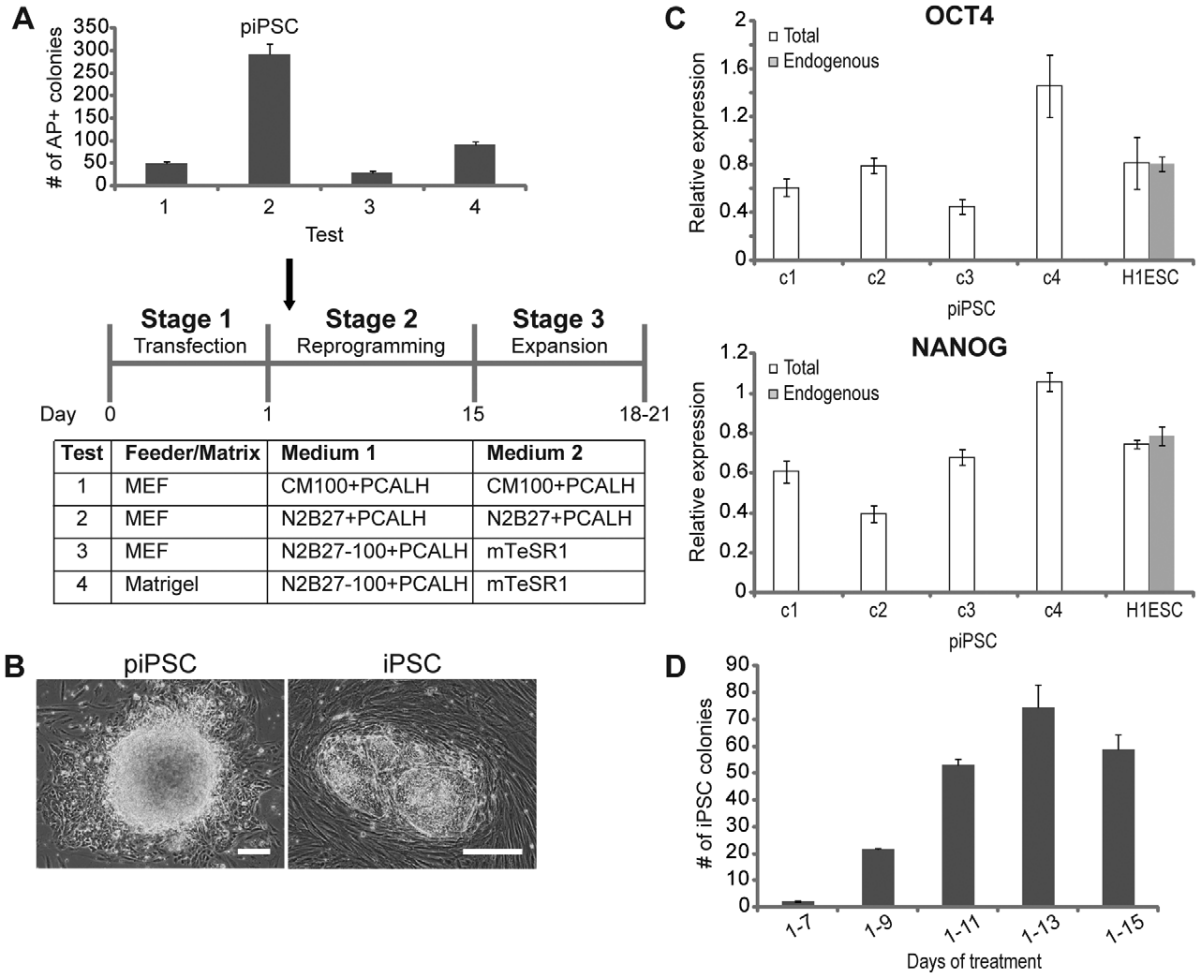
Developing a feeder-free condition for episomal reprogramming. (**A**) Effects of MEF feeder cells, matrigel and culture media on episomal reprogramming. Transfected human foreskin fibroblasts were plated to MEF feeder cell-seeded or matrigel-coated 10-cm dishes, and subjected to different reprogramming culture conditions. Alkaline phosphatase positive (AP+) colonies were counted on day 18–21 post-transfection. The number of AP+ colonies was from ∼0.33×10^6^ input cells. Data shown are mean ± SEM (n = 3). **N2B27**: DMEM/F12 medium supplemented with N-2 and B-27; **N2B27-100**: N2B27 medium supplemented with 100 ng/ml bFGF. (**B**) Bright-field images of a piPSC colony from test 2 and a typical human ESC-like iPSC colony from test 1, 3 or 4. Scale bars: 100 µm. (**C**) Quantitative RT-PCR analysis of OCT4 and NANOG expression in piPSC clone 1 to 4 (c1 to c4, p3). Total: both endogenous and transgene expression. Human H1 ESCs (H1ESC, p32) were used as a control. Data shown are mean ± SEM (n = 3). (**D**) Temporal requirement of small molecule treatment for feeder-free episomal reprogramming (test 4) (Fig. 2A). Transfected human foreskin fibroblasts were plated to matrigel-coated 10-cm dishes. PCALH was added to the N2B27-100 medium for different days at stage 2 of reprogramming. Alkaline phosphatase positive iPSC colonies were counted on day 22 post-transfection. The number of iPSC colonies was from ∼0.33×10^6^ input cells. Data shown are mean ± SEM (n = 3).

Since the MEF feeder cells and human ESC medium used for reprogramming likely contains small molecule-mitigating activities, it is possible to further improve episomal reprogramming by using matrix and defined culture media. Such defined reprogramming conditions will not only reduce the quality variations associated with feeder cells and KnockOut serum replacement in the human ESC medium, but also can be readily adapted to the production of clinical-grade human iPSCs. To find defined media that could support episomal reprogramming, we initially tested the N2B27 medium since it has a simple formulation and was able to support the proliferation of human ESCs when supplemented with cytokines [27]. As shown in Figure 2A, the N2B27 medium supplemented with small molecules gave rise to nearly 6-fold higher number of colonies stained positive for alkaline phosphatase (a human pluripotent stem cell marker) (test 2 vs. test 1). These colonies (piPSC for partially reprogrammed iPSCs) had a mouse ESC-like domed morphology, which differs from the flattened compact morphology typical of human ESC-like iPSC colonies (Fig. 2B). They could be expanded for more than 10 passages in the N2B27 medium supplemented with small molecules. Flow cytometry analysis of these cells, however, showed no expression of human ESC-specific cell surface antigens (SSEA-3, SSEA-4, Tra-1-60 and Tra-1-81), while the expression of a fibroblast marker CD44 was present (Fig. S2A). Quantitative RT-PCR analysis also failed to detect any expression of the endogenous *OCT4* and *NANOG*, two essential markers for human pluripotent stem cells (Fig. 2C). These results suggested that the colonies were partially reprogrammed iPSCs, not human ESC-like iPSCs as those derived with the human ESC medium (test 1) or mouse ESC-like iPSCs, thus illustrating the important influence of reprogramming culture conditions on the pluripotent state of iPSCs [28]–[29]. Interestingly, the piPSCs contained abundant episomal vectors and maintained high-level transgene expression even after multiple passages in the N2B27 medium supplemented with small molecules (Fig. 2C and Fig. S2B), suggesting a likely mechanism that these small molecules improved episomal reprogramming was through the retention of episomal vectors and transgene expression. Removal of small molecules led to occasional emergence of human ESC-like iPSCs after two weeks' culture of piPSCs in the chemically defined human ESC medium mTeSR1. Thus, although the N2B27 medium failed to yield authentic iPSCs, modifications could be made in the reprogramming protocol to enable the derivation of human ESC-like iPSCs using the N2B27 medium.

To this end, we divided the reprogramming process into three stages: transfection of human somatic cells with episomal reprogramming vectors (stage 1), reprogramming using N2B27 medium supplemented with small molecules (stage 2), and expansion with mTeSR1 medium (stage 3) (Fig. 2A). When the N2B27 medium supplemented with small molecules was used at stage 2 to support reprogramming, only rare conversion of piPSCs to human ESC-like iPSCs could be observed, suggesting that the transgene expression during expansion in mTeSR1 was insufficient to reactivate the expression of the endogenous pluripotent genes in most piPSCs. Thus we examined whether it was possible to improve episomal reprogramming by adding additional cytokines in the N2B27 medium supplemented with small molecules (stage 2). Of factors that are involved in the proliferation of human ESCs, bFGF and TGF-β/Activin/Nodal signaling are of particular importance. As inhibition of TGF-β/Activin/Nodal signaling by A-83-01 facilitated reprogramming (Fig. 1A), we tested the effect of bFGF on episomal reprogramming. Indeed, addition of high concentration bFGF to the N2B27 medium yielded reasonable number of human ESC-like iPSC colonies (test 3) (Fig. 2A). This result was consistent with previous observations that high concentration bFGF supported human ESC growth through multiple pathways besides MEK. Importantly, removal of MEF feeder cells by replacing with matrigel yielded even higher reprogramming efficiency (test 4) (Fig. 2A), consistent with the previous hypothesis that the MEF feeder cells contained small molecule-mitigating activities. Time-course experiments showed a requirement for an optimal time window of small molecule treatment (Fig. 2D). Thus, using small molecules and defined media (test 4) (Fig. 2A), we have established a feeder free episomal reprogramming method with significantly improved efficiency (∼220 iPSC colonies from 1×10^6^ input fibroblasts, ∼70-fold increase) (Fig. 2D).

### Characterizing iPSCs generated with the feeder-free small molecule-aided episomal reprogramming method

**Figure 3:**
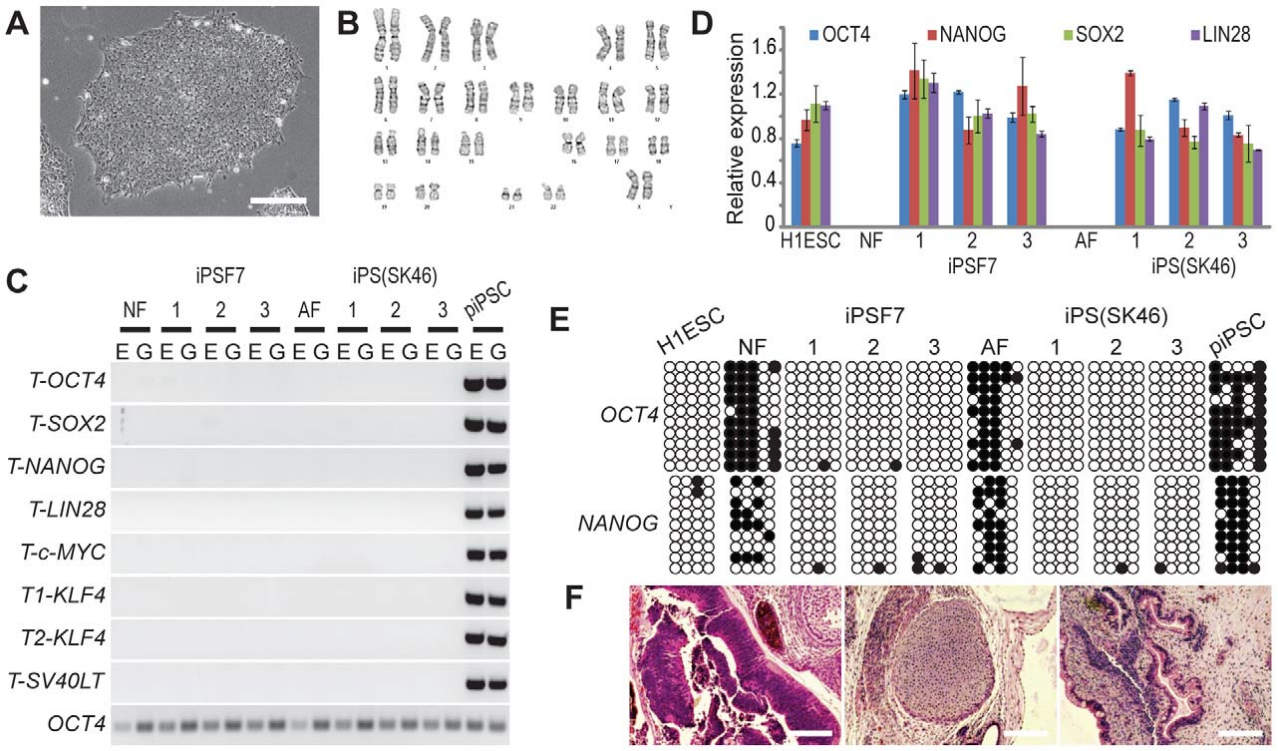
Characterization of iPSCs derived using the small molecule-aided feeder-free condition. (**A**) Bright-field image of iPSCs derived from human adult skin fibroblasts (iPS(SK46) clone 2). Scale bar: 100 µm. (**B**) G-banding chromosome analysis of iPS(SK46) clone 2 (p17). (**C**) PCR analysis of reprogramming vectors in iPSCs. E: episomal DNA; G: genomic DNA; NF: neonatal foreskin fibroblasts (p5); iPSF7 clone 1 to 3: iPSCs derived from neonatal foreskin fibroblasts (p26); AF: adult skin fibroblasts (p6); iPS(SK46) clone 1 to 3: iPSCs derived from adult skin fibroblasts (p22). piPSC derived from human foreskin fibroblasts (p4) were used as controls. *T-OCT4*: transgene *OCT4*; *T-SOX2*: transgene *SOX2*; *T-NANOG*: transgene *NANOG; T-LIN28*: transgene *LIN28*; *T-c-MYC*: transgene *c-MYC*; *T1-KLF4*: transgene *KLF4 (1)*; *T2-KLF4*: transgene *KLF4 (2)*; *T-SV40LT*: transgene *SV40LT*; *OCT4*: endogenous *OCT4*. 32 PCR cycles were used for all primer sets. (**D**) Quantitative RT-PCR analysis of the endogenous OCT4, NANOG, SOX2 and LIN28 expression in iPSC clones. Data shown are mean ± SEM (n = 3). (**E**) Bisulfite-sequencing analysis of the methylation status of the *OCT4* and *NANOG* promoters in iPSC clones. Open circles indicate unmethylated, and filled circles indicate methylated CpG dinucleotides. (**F**) Hematoxylin and eosin staining of teratoma sections of iPSC(SK46) clone 2. Teratomas were obtained from all iPSC clones. Left panel: neural tissue (ectoderm); middle panel: cartilage (mesoderm); right panel: gut epithelium (endoderm). Scale bars: 100 µm.

With the newly developed feeder-free reprogramming condition, we have successfully derived human ESC-like iPSCs from neonatal and adult skin fibroblasts. When picked and expanded in mTeSR1, these iPSCs showed typical human ESC morphology (e.g. compact colonies, high nucleus-to-cytoplasm ratios and prominent nucleoli), and had normal karyotypes (Fig. 3 and Fig. S3). Most iPSC colonies showed no transgene expression or genomic integration, and had completely lost episomal vectors after about 14 passages as demonstrated by PCR and RT-PCR analysis (Fig. 3C and Fig. S3C). They expressed typical human ESC-specific antigens (SSEA-3, SSEA-4, Tra-1-60 and Tra-1-81), down-regulated the expression of the fibroblast marker CD44 (Fig. S3D), and reactivated the expression of the endogenous pluripotent genes (*OCT4*, *NANOG*, *SOX2* and *LIN28*) (Fig. 3D). Both the *OCT4* and *NANOG* promoters were demethylated in these iPSCs, similar to human ESCs and in contrast to the parental fibroblasts and piPSCs (Fig. 3E). When injected into immunocompromised mice, they formed teratomas consisting of derivatives of all three germ layers, demonstrating the pluripotency of these iPSCs (Fig. 3F and Fig. S3E).

### Episomal reprogramming of different human somatic cell types

**Figure 4:**
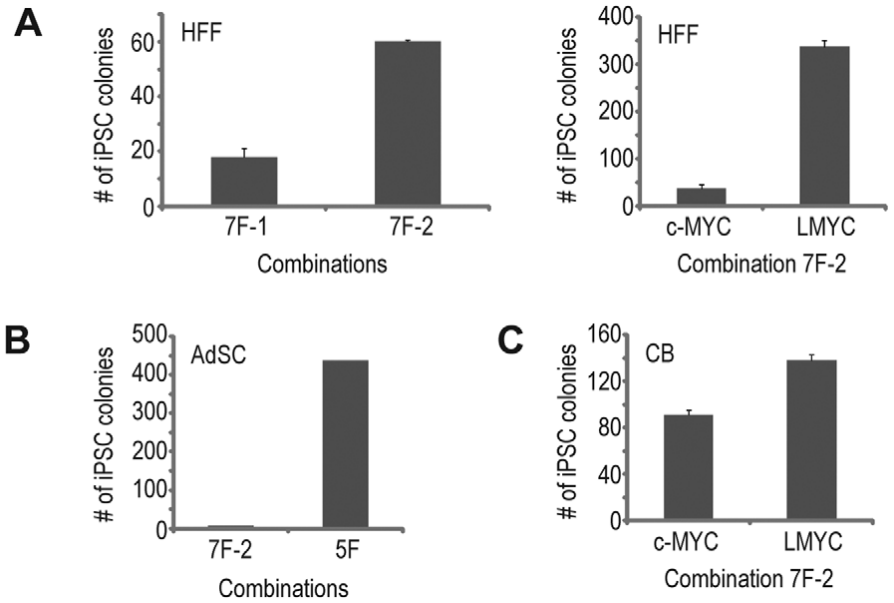
Optimizing episomal vector and transgene combinations for small molecule-aided reprogramming of different human somatic cells. (**A**) Effects of different episomal vector combinations and transformation-deficient LMYC on the reprogramming of human fibroblasts. Transfected human foreskin fibroblasts (HFFs) were plated to matrigel-coated 10-cm dishes. N2B27-100 medium supplemented with PCALH was used to support reprogramming between day 2 and 13 post-transfection, followed by mTeSR1 for expansion. The number of iPSC colonies (from ∼0.33×10^6^ input cells) was counted on day 21 post-transfection. Data shown are mean ± SEM (n = 3). (**B**) Effects of different transgene combinations on the reprogramming of human adult adipose tissue-derived stem cells (AdSCs). Transfected adipose tissue-derived stem cells were plated to matrigel-coated 10-cm dishes. N2B27-100 medium supplemented with PCALH was used to support reprogramming between day 2 and 11 post-transfection, followed by mTeSR1 for expansion. The number of iPSC colonies (from ∼0.35×10^6^ input cells) was counted on day 21 post-transfection. (**C**) Effects of transformation-deficient LMYC on the reprogramming of human cord blood (CB) cells. Purified human cord blood CD34+ cells were expanded in culture for 7 days (∼75 fold expansion). Following expansion, the cord blood cells were nucleofected with episomal vectors and plated to fibronectin/matrigel-coated 6-well plates. N2B27-100 medium supplemented with PCALH was used to support reprogramming between day 2 and 11 post-transfection, followed by mTeSR1 for expansion. The number of iPSC colonies (from ∼0.17×10^6^ post-expansion input cells) was counted on day 17 post-transfection. Data shown are mean ± SEM (n = 6).

Using human fibroblasts, we have successfully established a feeder-free small molecule-aided episomal reprogramming method. Though the reprogramming efficiency was high enough to enable routine iPSC derivation from human adult fibroblasts, we sought to further improve the efficiency by modifying the episomal vectors. Our previous work demonstrated that the balance between the expression of different transgenes had great impact on the reprogramming efficiency [19]. Since the transgene expression from different episomal vectors differs, we tested the two episomal vector combinations that were previously shown to be functional [19] (Fig. S1A and S1B): a two-vector combination 7F-1, which was used for the studies above, and a three-vector combination 7F-2. Both combinations contain the expression cassettes for all seven transgenes (*OCT4*, *SOX2*, *NANOG*, *LIN28*, *c-MYC*, *KLF4* and *SV40LT*). As shown in Figure 4A, the three-vector combination 7F-2 consistently yielded more iPSC colonies than the two-vector combination 7F-1. More importantly, the replacement of *c-MYC* in the three-vector combination 7F-2 with transformation-deficient *LMYC* further improved the episomal reprogramming efficiency (∼1000 iPSC colonies from 1×10^6^ input fibroblasts) (Fig. 4A), which is consistent with earlier studies [30]. Thus with *LMYC*, the small molecule-aided episomal reprogramming method is robust for human skin fibroblasts.

Since skin fibroblast might contain higher number of somatic mutations, they might not be an ideal cell type for the production of clinical-grade human iPSCs. We tested episomal reprogramming of additional cell types that are readily available from human donors, specifically, adipose tissue derived cells and cord blood cells. As shown in Figure 4B and 4C, the small molecule-aided episomal reprogramming method could be easily adapted to these two cell types. For adipose tissue-derived cells, the three-vector combination 7F-2 only yielded very few iPSCs, although many piPSC colonies were present. Removal of *c-MYC* and *KLF4* (combination 5F: *OCT4*, *SOX2*, *NANOG*, *LIN28* and *SV40LT*), however, gave rise to numerous iPSC colonies (∼1200 iPSC colonies from 1×10^6^ input human adult adipose tissue-derived cells) (Fig. 4B), thus stressing the importance of finding the right transgene combination for each cell type in order to achieve the optimal reprogramming efficiency. Cord blood cells, compared to other somatic cell types, have unique advantages as donor cells for the production of clinical-grade human iPSCs, since these cells potentially contain less somatic mutations, and are readily available. With the small molecule-aided episomal reprogramming method, high reprogramming efficiency was also achieved with these cells (∼800 iPSC colonies from 1×10^6^ input post-expansion cord blood cells, i.e. ∼600 iPSC colonies per 1×10^4^ input freshly isolated cord blood CD34+ cells with 75-fold expansion during 7-day culture) (Fig. 4C).

## Discussion

In this report, using a combined genetic and chemical approach, we have successfully established a robust feeder-free episomal reprogramming method using defined media. Though developed with fibroblasts, this method was readily adapted to adipose tissue-derived cells, cord blood cells, and potentially other cell types easily accessible from human donors. Additional features can be introduced into episomal vectors to further improve reprogramming efficiency. For example, the current episomal vectors have elements necessary for bacterial propagation, which contain many CpG islands known to contribute to transgene silencing [31]. It is possible to minimize transgene silencing by removing the bacterial vector component using site-specific recombination to produce minicircle oriP/EBNA-1 episomal vectors. Nevertheless, the current method is robust enough for the routine derivation of footprint-free iPSCs from different somatic cell types and from many human donor samples.

Selection of the right episomal vector combination and transgene combination appeared to be important for achieving optimal reprogramming efficiency for different somatic cell types (Fig. 4A and 4B), likely reflecting their different requirement of balanced transgene expression. Since only a single transfection is needed for episomal reprogramming, this method could be readily applied to many cell types including blood cells such as cord blood cells, although peripheral blood cells might represent an ideal cell source for large-scale studies as established procedures are readily available for blood handling (e.g. collection, cryopreservation, shipping and cell isolation). It is interesting to see how well this method can be applied to peripheral blood cells, particularly cells without genomic rearrangements. The current episomal reprogramming method used matrigel as the matrix to support reprogramming and iPSC expansion due to its affordability. This, however, introduced undefined components into the method. For the production of clinical-grade human iPSCs, the matrigel can most likely be replaced with defined matrix that supports both donor cell and iPSC growth, such as vitronectin [32]. The disadvantage with the episomal method, compared to mRNA-mediated reprogramming [17], is that the iPSCs will need to undergo multiple passages to completely lose episomal vectors (e.g. ∼14 passages for fibroblast-derived iPSCs). This, however, will unlikely present a problem in applications, since iPSCs will need to undergo multiple passages to be rid of epigenetic memory of the donor cells [18], and to be fully characterized for downstream applications.

Episomal reprogramming in the presence of small molecules appeared to progress through a distinct intermediate stage - piPSCs (Fig. 2B), as the same cells were obtained when the N2B27 medium was supplemented with bFGF in test 2 (Fig. 2A). These piPSCs expressed alkaline phosphatase, could be continuously passaged in the presence of small molecules, and retained episomal reprogramming vectors, transgene expression and the ability to give rise to human ESC-like iPSCs upon withdrawal of small molecules. The generation of these piPSCs depended on both the SV40LT expression and the small molecule treatment, since none of these piPSCs could be observed in the absence of the SV40 LT expression or the small molecules. In our previous report, we included SV40LT to counteract the toxic effects caused by c-MYC overexpression [19]. The role of SV40LT in reprogramming, however, is rather controversial. It was initially used in combination with OCT4, SOX2, c-MYC and KLF4 to reprogram human fibroblasts, but was found to be absent from the iPSCs generated [33]. This suggested that the SV40LT expression did not contribute to iPSC derivation. A second report showed that the SV40LT expression enhanced both the reprogramming efficiency and the reprogramming pace [34]. Results from our own work showed that although the SV40LT expression greatly facilitated the appearance of reprogramming intermediates, its continuous expression blocked the iPSC generation (unpublished data). With the small molecule-aided episomal reprogramming in defined media, the SV40LT expression seemed critical, since no piPSCs or iPSCs were obtained from fibroblasts in its absence. Truncated mutants of SV40LT such as its transformation domain (N-terminal 147 amino acids) and p53-binding domain failed to replace the full-length SV40LT (unpublished data). It remains an intriguing question how the combination of the SV40LT expression and small molecule treatments enables the prolonged retention of oriP/EBNA-1-based reprogramming vectors and transgene expression. More importantly, due to this distinct reprogramming intermediate stage, it will be of great interest to characterize the iPSCs obtained with this method in details, e.g. the mutation rate arising during reprogramming and the epigenetic memory retention. This will help address the question whether the quality of iPSCs is significantly affected by their derivation methods.

In summary, using defined media, we have successfully established a robust feeder-free episomal reprogramming method, which applies to different somatic cell types readily available from human donors. This method can be used for the derivation of footprint-free iPSCs from many donor samples, and can be easily adapted to the production of clinical-grade human iPSCs.

## Materials and Methods

### Ethics Statement

All human primary cells were generated in vitro from tissue samples from human donors with appropriate written informed consent given to the commercial providers.

All mouse work was conducted according to relevant national and international guidelines under the approval of the Cellular Dynamics International Animal Care and Use Committee. As a private company, our animal facility does not provide a permit number or approval ID since mouse is not a protected species.

### Cell culture

Human ESCs and iPSCs were maintained on irradiated MEFs in DMEM/F12 culture medium supplemented with 20% KnockOut serum replacement, 0.1 mM non-essential amino acids, 1 mM GlutaMAX (all from Invitrogen, Carlsbad, CA), 0.1 mM β-mercaptoethanol (Sigma, St. Louis, MO) and 100 ng/ml zebrafish basic fibroblast growth factor (zbFGF) [19]. MEF-conditioned human ESC medium was prepared as previously described [35]. Human newborn foreskin fibroblasts (Cat# CRL-2097, ATCC, Manassas, MA) and adult skin fibroblasts (Cat# CRL-2106, ATCC) were cultured in DMEM (Invitrogen) supplemented with 10% heat-inactivated fetal bovine serum (FBS, HyClone Laboratories, Loan, UT), 0.1 mM non-essential amino acids, 1 mM GlutaMAX, 0.1 mM β-mercaptoethanol and 4 ng/ml zbFGF. Human adult adipose tissue-derived stem cells (Cat# ASC-F, ZenBio, Research Triangle Park, NC) was cultured in MesenCult-XF basal medium supplemented with 1×MesenCult-XF supplement (STEMCELL Technologies, Vancouver, BC, Canada) and 2 mM GlutaMAX on collagen (60 µg per 10-cm dish)/fibronectin (18 µg per 10-cm dish)-coated 10-cm dishes. Human cord blood CD34+ cells were obtained from STEMCELL Technologies (Cat# CB008F-S), and expanded on fibronectin-coated plates in CD34+ cell expansion medium - StemSpan SFEM (STEMCELL Technologies) supplemented with EX-CYTE growth enhancement media supplement (1∶1000, Millipore, Billerica, MA), 2 mM GlutaMAX, 250 ng/ml SCF, 250 ng/ml FLT3L, 100 ng/ml TPO, 20 ng/ml IL-3, 50 ng/ml IL-6 and 10 ng/ml sIL6-R (all from Peprotech, Rocky Hill, NJ).

The feeder-free culture of human ESCs and iPSCs on matrigel (BD Biosciences, Bedford, MA) in mTeSR1 (STEMCELL Technologies) was carried out as previously described with modifications in the passaging procedure [36]. Briefly EDTA splitting method was employed. When human ESCs and iPSCs reach confluence, cells were washed once with PBS free of Ca^2+^ and Mg^2+^, and incubated with 0.5 mM EDTA for 8 minutes at 37°C (2 ml/well of 6-well plate). After incubation, the EDTA solution was removed and fresh mTeSR1 (2 ml/well of 6-well plate) was added dropwise to each well for cell detachment. Most cells came off the plate with gentle shaking. Dissociated cells were then immediately dispersed into freshly prepared matrigel plates prefilled with mTeSR1. To improve cell attachment and survival, the ROCK inhibitor HA-100 (10 µM, Santa Cruz Biotechnology, Santa Cruz, CA) was added to mTeSR1 for 1 day during passaging. With this method, human ESCs and iPSCs were passaged every 3 to 4 days at a splitting ratio of 1∶8 for optimal growth.

### Improve episomal reprogramming of human fibroblasts with small molecules

Episomal reprogramming vectors containing expression cassettes for human *OCT4*, *SOX2*, *NANOG*, *LIN28*, *c-MYC*, *KLF4* and *SV40LT* transgenes were as previously described [19]. Specifically, vector pEP4EO2SCK2MEN2L and pEP4EO2SET2K (combination 7F-1, Fig. S1A) were used for reprogramming optimization. About 7.3 µg of vector pEP4EO2SCK2MEN2L and 3.2 µg of pEP4EO2SET2K were co-transfected into ∼1.0×10^6^ human neonatal foreskin fibroblasts via nucleofection (VPD-1001 with program U-20, Amaxa, Walkersville, MD). Transfected fibroblasts were plated directly to 3×10-cm MEF-seeded or matrigel-coated dishes in fibroblast culture medium. On day one post-transfection, the fibroblast medium was replaced with MEF-conditioned human ESC medium supplemented with 100 ng/ml zbFGF (CM100), or chemically defined N2B27 medium (N2B27), or N2B27 medium supplemented with 100 ng/ml zbFGF (N2B27-100). The N2B27 medium consists of DMEM/F12 culture medium supplemented with N-2 supplement (1×, Invitrogen), B-27 supplement (1×, Invitrogen), 0.1 mM non-essential amino acids, 1 mM GlutaMAX, and 0.1 mM β-mercaptoethanol. Where applied, small molecules PD0325901 (P, 0.5 µM), CHIR99021(C, 3 µM), A-83-01(A, 0.5 µM) (all from Stemgent, San Diego, CA), hLIF (L, 1000 U/ml, Millipore) and HA-100 (H, 10 µM) were added to reprogramming culture. Culture medium was refreshed every two days. Alkaline phosphatase staining (Cat# SCR004, Millipore) was performed in subsets of reprogramming experiments to facilitate the identification of iPSCs. Briefly, cells were fixed in 2% paraformaldehyde (Electron Microscopy Sciences, Hatfield, PA) for 30 minutes at room temperature before staining according to manufacturer's protocol. Authentic iPSC colonies were stained positive for alkaline phosphatase, and showed morphology typical of human ESCs (e.g. compact colonies, high nucleus-to-cytoplasm ratios and prominent nucleoli) (Fig. S4). Episomal reprogramming of human adult skin fibroblasts was carried out similarly to that of foreskin fibroblasts. To characterize the newly derived iPSCs, iPSC colonies were picked directly onto matrigel-coated 12-well plates in mTeSR1. EDTA splitting method was employed to facilitate iPSC expansion and removal of differentiated cells. Complete loss of episomal vectors was generally achieved around passage 14 for fibroblast-derived iPSC clones.

### Optimizing episomal vector and transgene combinations for reprogramming human fibroblasts, adult adipose tissue-derived cells and cord blood cells

Episomal reprogramming vector combinations tested were shown in Figure S1. For each nucleofection, the following amount of DNA was used: combination **7F-1** (7.3 µg of pEP4EO2SCK2MEN2L and 3.2 µg of pEP4EO2SET2K); combination **7F-2** (3.0 µg of pEP4EO2SEN2K, 3.2 µg of pEP4EO2SET2K and 2.4 µg of pCEP4-M2L); combination **5F** (2.8 µg of pEP4EO2SEN2L and 3.2 µg of pEP4EO2SET2N). To test the effect of transformation-deficient *LMYC* on episomal reprogramming, the coding region of *LMYC* was cloned via PCR from human ESCs and used to replace the coding region of *c-MYC* in the vector pCEP4-M2L of combination **7F-2**.

Reprogramming of human foreskin fibroblasts was carried out as described above. Briefly transfected fibroblasts (∼1.0×10^6^ cells per nucleofection) were plated directly to 3×10-cm matrigel-coated dishes in fibroblast culture medium. On day 2 post-transfection, the fibroblast medium was replaced with chemically defined N2B27-100 supplemented with PCALH. Culture medium was refreshed every two days. mTeSR1 was used to expand iPSCs on day 13 post-transfection. The total number of iPSC colonies was counted on day 21 post-transfection.

Reprogramming of human adult adipose tissue-derived cells was carried out similarly to that of human fibroblasts. Briefly, nucleofected adipose tissue-derived cells (VPE-1001 with program A-33, Amaxa, ∼0.7×10^6^ cells per nucleofection) were plated directly to 2×10-cm matrigel-coated dishes in fresh adipose tissue-derived cell culture medium. N2B27-100 supplemented with PCALH was used to support reprogramming between day 2 and 11 post-transfection followed by mTeSR1 for iPSC expansion. The total number of iPSC colonies was counted on day 21 post-transfection.

Reprogramming of cord blood cells was similar to that of fibroblasts and adipose tissue-derived cells. Due to the limited number of cord blood CD34+ cells and to improve the initial cell survival following nucleofection, frozen CD34+ cells from STEMCELL Technologies were thawed and expanded in the CD34+ cell expansion medium for about 7 days. The CD34+ cells showed robust growth in culture (∼75 fold expansion). Following expansion, about 1×10^6^ cells were transfected with episomal vectors via nucleofection (VPA-1003 with program T-16, Amaxa), and plated to one fibronectin/matrigel-coated 6-well plate in CD34+ cell expansion medium. The fibronectin coating was used together with matrigel to facilitate the cord blood cell recovery from nucleofection. N2B27-100 supplemented with PCALH was used to support reprogramming between day 2 and 11 post-transfection followed by mTeSR1 for iPSC expansion. The total number of iPSC colonies was counted on day 17 post-transfection.

### RT-PCR expression analysis, PCR analysis of episomal vectors, bisulfite-sequencing analysis, flow cytometry analysis and karyotyping

PCR, RT-PCR, flow cytometry analysis were performed as previously described [2], [19]. For PCR analysis, episomal DNA was purified from human cells using QIAprep® Spin Miniprep Kit (Qiagen, Valencia, CA) with Proteinase K (NEB, Ipswich, MA) digestion as previously described [37]. Genomic DNA was isolated with the traditional phenol/chloroform extraction method. All the PCR reactions were carried out with pfx DNA polymerase (Invitrogen) with the amplification buffer used at 2X and the enhancer solution used at 3X, and with the following program: initial denaturation for 5 minutes at 94°C; 32 cycles of 94°C for 15 seconds, 55°C for 30 seconds, 68°C for 1 minute; and followed by 68°C for 7 minutes. For each PCR reaction, the episomal DNA from 1×10^4^ cells equivalent or about 0.1 µg genomic DNA was added as template. Episomal DNA and genomic DNA from nontransfected fibroblasts were used as negative controls, while those from piPSCs were used as positive controls. For RT-PCR analysis, total RNA was prepared as described in the RNeasy Mini Kit (Qiagen) with on-column DNase I digestion. About 1 µg total RNA from each sample was used for Oligo(dT)_20_-primed reverse transcription as described in the product protocol (SuperScript™ III First-Strand Synthesis System, Invitrogen). The PCR reactions for regular RT-PCR analysis of transgene expression were carried out with the pfx DNA polymerase (Invitrogen) as described above. The cDNA from nontransfected fibroblasts and human H1 ESCs were used as negative controls, while that from piPSCs was used as positive controls. For each sample, 2 µl of diluted cDNA (1∶4) was added as template in PCR reactions. Quantitative PCR reactions were carried out with Power SYBR®Green PCR Master Mix (LightCycler® 480 II system, Roche, Indianapolis, IN). The cDNA from human H1ESC was used as a relative standard. For each sample, 1 µl of diluted cDNA (1∶8) was added as template in PCR reactions. The expression of genes of interest was normalized to that of *GAPDH* in all samples. The methylation status of *OCT4* and *NANOG* promoters were analyzed using bisulfite sequencing with MethylCode™ Bisulfite Conversion Kit (Invitrogen) [19]. All primers were in Table S1 and antibodies were in Table S2. Standard G-banding chromosome analysis was carried out in the Cytogenetics Lab at WiCell Research Institute (Madison, WI).

### Teratoma formation

To examine the *in vivo* differentiation potential of human iPSCs derived with the new episomal method, iPSCs grown on matrigel in mTeSR1 were transferred to MEF feeder cells for one passage. Cells were collected with collagenase treatment, and injected into hind limb muscles of 6-week-old immunocompromised SCID-beige mice (one 10-cm dish with 50 to 80% confluence per injection per mouse) (Harlan, Madison, WI). Two injections were performed for each iPSC clone. After six to eight weeks, teratomas were obtained from all injections, which were similar in size as those from human ESCs and iPSCs generated with previous methods. The teratomas were dissected and fixed in 10% formalin (Fisher, Pittsburgh, PA). Samples were embedded in paraffin and processed with hematoxylin and eosin staining in the Experimental Pathology Department of McArdle Laboratory for Cancer Research, University of Wisconsin-Madison, WI.

## Supporting Information

Figure S1 **Episomal reprogramming vector maps.** (**A**) Combination 7F-1 contains two vectors: pEP4EO2SCK2MEN2L and pEP4EO2SET2K. This combination expresses all seven transgenes: *OCT4*, *SOX2*, *NANOG*, *LIN28*, *c-MYC*, *KLF4* and *SV40LT*. (**B**) Combination 7F-2 contains three vectors: pEP4EO2SEN2K, pEP4EO2SET2K and pCEP4-M2L. This combination also expresses all seven transgenes: *OCT4*, *SOX2*, *NANOG*, *LIN28*, *c-MYC*, *KLF4* and *SV40LT*. (**C**) Combination 5F contains two vectors: pEP4EO2SEN2L and pEP4EO2SET2N. This combination expresses five transgenes: *OCT4*, *SOX2*, *NANOG*, *LIN28* and *SV40LT*. pEF: the eukaryotic elongation 1α promoter; pCMV: the cytomegalovirus immediate-early promoter; IRES2: internal ribosome entry site 2.
(TIF)

Figure S2 **Developing a feeder-free condition for episomal reprogramming.** (**A**) Flow cytometry expression analysis of human ESC-specific cell surface markers (SSEA-3, SSEA-4, Tra-1-60 and Tra-1-81) and a fibroblast marker CD44 in piPSCs (p6). Unfilled: isotype control; filled: antigen staining. (**B**) PCR analysis of reprogramming vectors in the episomal DNA isolated from piPSCs (p7). Lane 1: transgene *OCT4 (T-OCT4)*; Lane 2: transgene *NANOG (T-NANOG)*; Lane 3: transgene *KLF4 (1) (T1-KLF4)*; Lane 4: transgene *KLF4 (2) (T2-KLF4)*; Lane 5: transgene *SV40LT (T-SV40LT)*; Lane 6: transgene *SOX2 (T-SOX2)*; Lane 7: transgene *LIN28 (T-LIN28)*; Lane 8: transgene *c-MYC (T-c-MYC)*; Lane 9: endogenous *OCT4 (OCT4)*.
(TIF)

Figure S3 **Characterization of iPSCs derived using the small molecule-aided feeder-free condition.** (**A**) Bright-field image of iPSCs derived from human foreskin fibroblasts (iPSF7 clone 1). Scale bar: 100 µm. (**B**) G-banding chromosome analysis of iPSF7 clone 1 (p18). (**C**) RT-PCR analysis of transgene expression in iPSC clones. NF: neonatal foreskin fibroblasts (p5); iPSF7 clone 1 to 3: iPSCs derived from neonatal foreskin fibroblasts (p26); AF: adult skin fibroblasts (p6); iPS(SK46) clone 1 to 3: iPSCs derived from adult skin fibroblasts (p22). H1ESC (p32) and piPSC (p4) derived from human foreskin fibroblasts were used as controls. T-OCT4: transgene OCT4; T-SOX2: transgene SOX2; T-NANOG: transgene NANOG; T-LIN28: transgene LIN28; T-c-MYC: transgene c-MYC; T1-KLF4: transgene KLF4 (1); T2-KLF4: transgene KLF4 (2); T-SV40LT: transgene SV40LT; OCT4: endogenous OCT4; GAPDH: endogenous control. 32 PCR cycles were used for all primer sets except for T-OCT4 (30 cycles). (**D**) Flow cytometry expression analysis of human ESC-specific cell surface markers (SSEA-3, SSEA-4, Tra-1-60 and Tra-1-81) and the fibroblast-enriched marker CD44. Unfilled: isotype control; filled: antigen staining. (**E**) Hematoxylin and eosin staining of teratoma sections of iPSF7 clone 1. Top panel: neural tissue (ectoderm); middle panel: cartilage (mesoderm); bottom panel: gut epithelium (endoderm). Scale bars: 100 µm.
(TIF)

Figure S4 **Bright-field (BF) images of an intermediate-stage colony (non-iPSC) and an iPSC colony stained positive for alkaline phosphatase.** These were typical colonies observed when fibroblasts were episomally reprogrammed with small molecule-supplemented CM100 on MEF feeder cells.
(TIF)

Table S1 **Primers for PCR, RT-PCR and bisulfite-sequencing PCR.**
(DOC)

Table S2 **Antibodies for flow cytometry analysis.**
(DOC)
