# Supplementary figures and images for: Efficient Feeder-Free Episomal Reprogramming with Small Molecules
Source: PLoS One. 2011 Mar 1;6(3):e17557. doi: 10.1371/journal.pone.0017557 (PMC3046978; doi:10.1371/journal.pone.0017557)

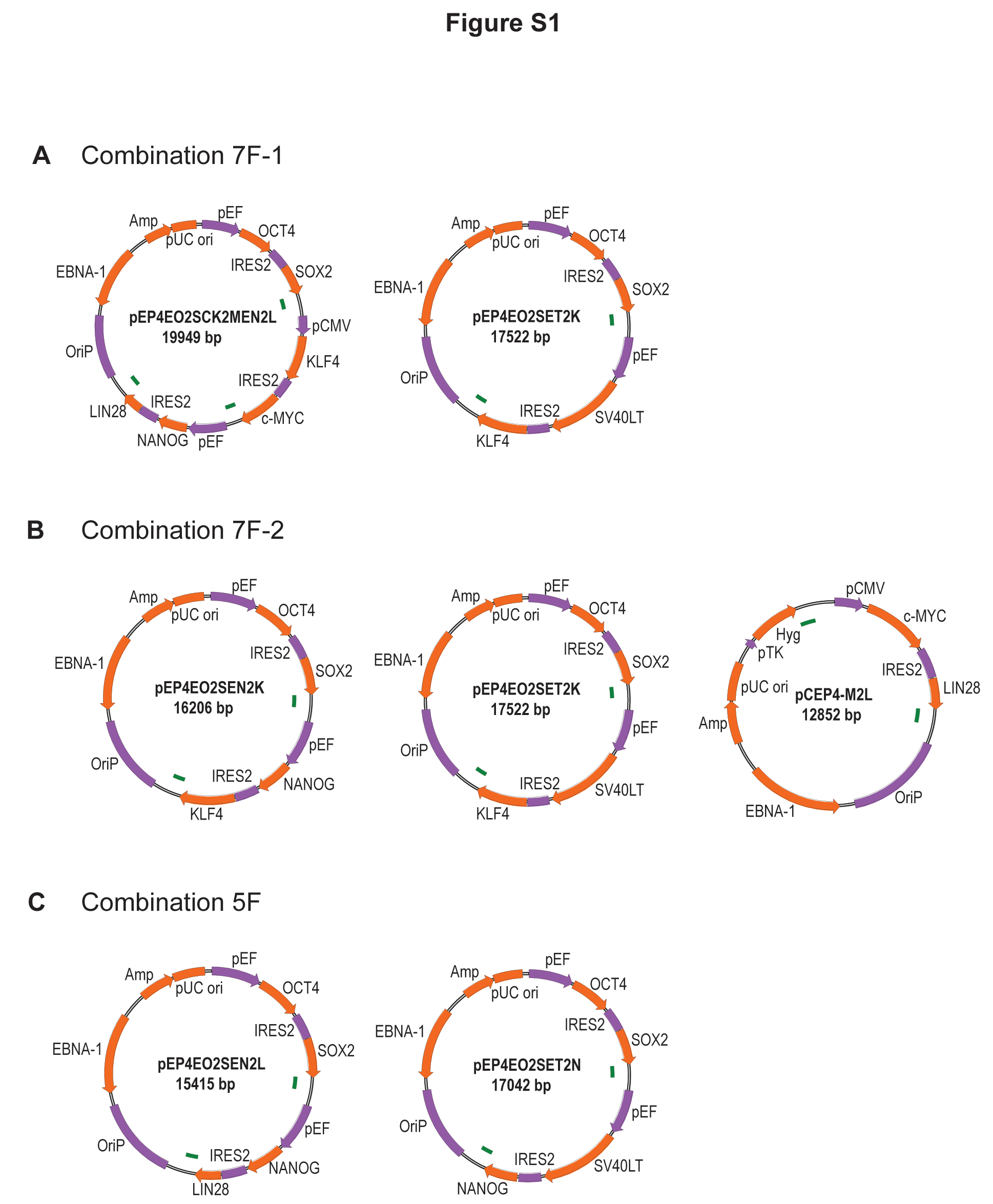

Supplement: Figure S1 — Episomal reprogramming vector maps. (A) Combination 7F-1 contains two vectors: pEP4EO2SCK2MEN2L and pEP4EO2SET2K. This combination expresses all seven transgenes: OCT4, SOX2, NANOG, LIN28, c-MYC, KLF4 and SV40LT. (B) Combination 7F-2 contains three vectors: pEP4EO2SEN2K, pEP4EO2SET2K and pCEP4-M2L. This combination also expresses all seven transgenes: OCT4, SOX2, NANOG, LIN28, c-MYC, KLF4 and SV40LT. (C) Combination 5F contains two vectors: pEP4EO2SEN2L and pEP4EO2SET2N. This combination expresses five transgenes: OCT4, SOX2, NANOG, LIN28 and SV40LT. pEF: the eukaryotic elongation 1α promoter; pCMV: the cytomegalovirus immediate-early promoter; IRES2: internal ribosome entry site 2. (TIF) [file pone.0017557.s001.tif]

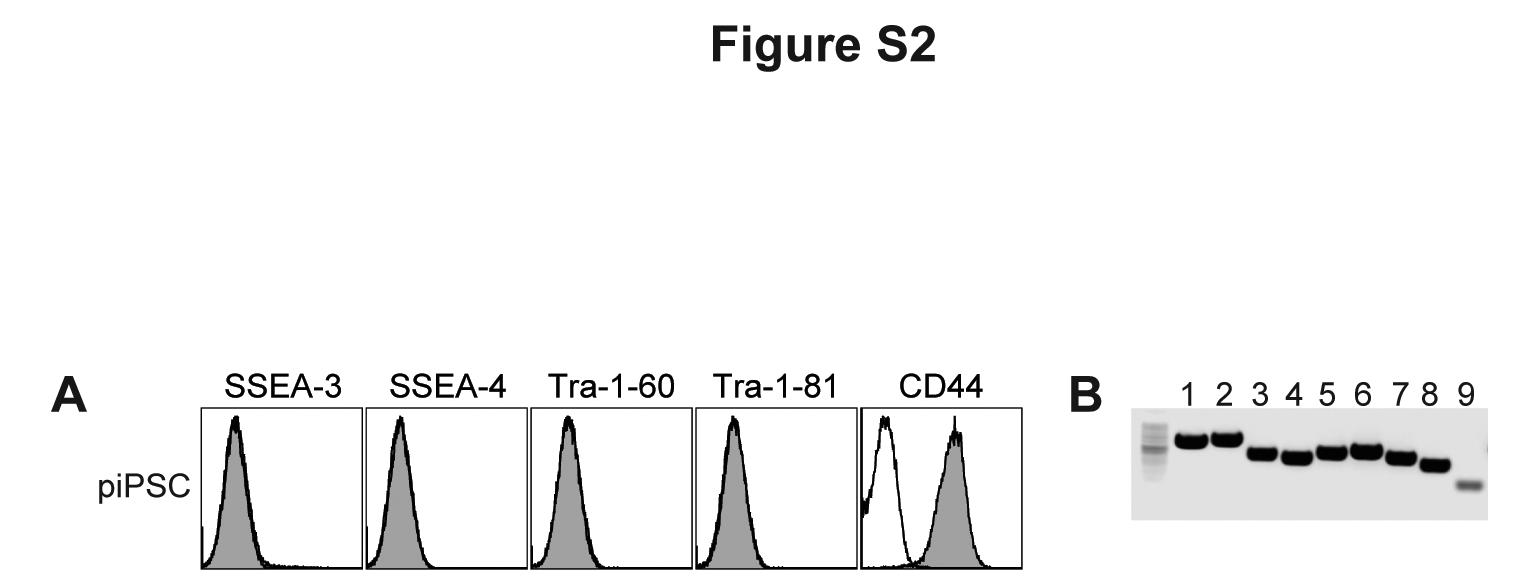

Supplement: Figure S2 — Developing a feeder-free condition for episomal reprogramming. (A) Flow cytometry expression analysis of human ESC-specific cell surface markers (SSEA-3, SSEA-4, Tra-1-60 and Tra-1-81) and a fibroblast marker CD44 in piPSCs (p6). Unfilled: isotype control; filled: antigen staining. (B) PCR analysis of reprogramming vectors in the episomal DNA isolated from piPSCs (p7). Lane 1: transgene OCT4 (T-OCT4); Lane 2: transgene NANOG (T-NANOG); Lane 3: transgene KLF4 (1) (T1-KLF4); Lane 4: transgene KLF4 (2) (T2-KLF4); Lane 5: transgene SV40LT (T-SV40LT); Lane 6: transgene SOX2 (T-SOX2); Lane 7: transgene LIN28 (T-LIN28); Lane 8: transgene c-MYC (T-c-MYC); Lane 9: endogenous OCT4 (OCT4). (TIF) [file pone.0017557.s002.tif]

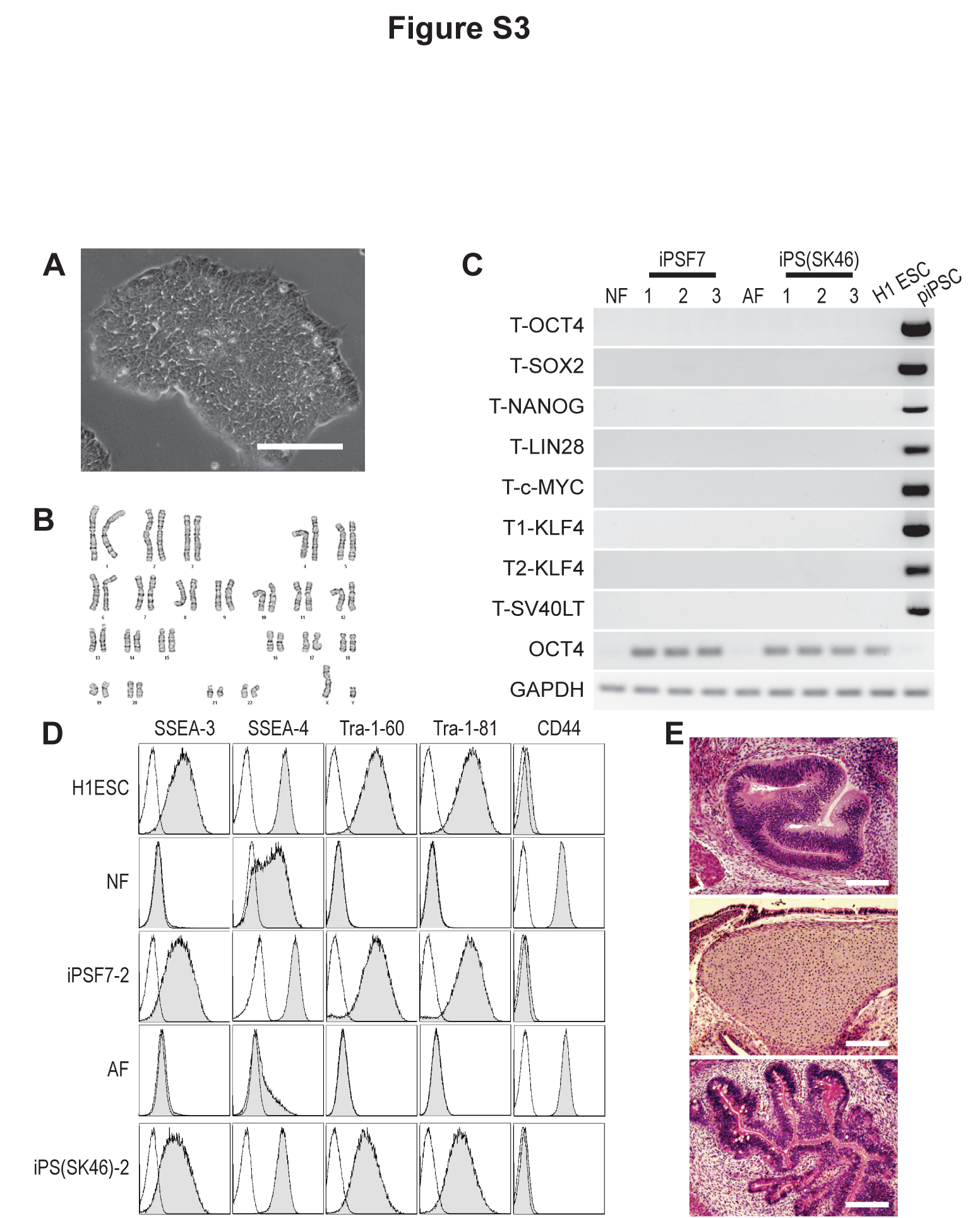

Supplement: Figure S3 — Characterization of iPSCs derived using the small molecule-aided feeder-free condition. (A) Bright-field image of iPSCs derived from human foreskin fibroblasts (iPSF7 clone 1). Scale bar: 100 µm. (B) G-banding chromosome analysis of iPSF7 clone 1 (p18). (C) RT-PCR analysis of transgene expression in iPSC clones. NF: neonatal foreskin fibroblasts (p5); iPSF7 clone 1 to 3: iPSCs derived from neonatal foreskin fibroblasts (p26); AF: adult skin fibroblasts (p6); iPS(SK46) clone 1 to 3: iPSCs derived from adult skin fibroblasts (p22). H1ESC (p32) and piPSC (p4) derived from human foreskin fibroblasts were used as controls. T-OCT4: transgene OCT4; T-SOX2: transgene SOX2; T-NANOG: transgene NANOG; T-LIN28: transgene LIN28; T-c-MYC: transgene c-MYC; T1-KLF4: transgene KLF4 (1); T2-KLF4: transgene KLF4 (2); T-SV40LT: transgene SV40LT; OCT4: endogenous OCT4; GAPDH: endogenous control. 32 PCR cycles were used for all primer sets except for T-OCT4 (30 cycles). (D) Flow cytometry expression analysis of human ESC-specific cell surface markers (SSEA-3, SSEA-4, Tra-1-60 and Tra-1-81) and the fibroblast-enriched marker CD44. Unfilled: isotype control; filled: antigen staining. (E) Hematoxylin and eosin staining of teratoma sections of iPSF7 clone 1. Top panel: neural tissue (ectoderm); middle panel: cartilage (mesoderm); bottom panel: gut epithelium (endoderm). Scale bars: 100 µm. (TIF) [file pone.0017557.s003.tif]

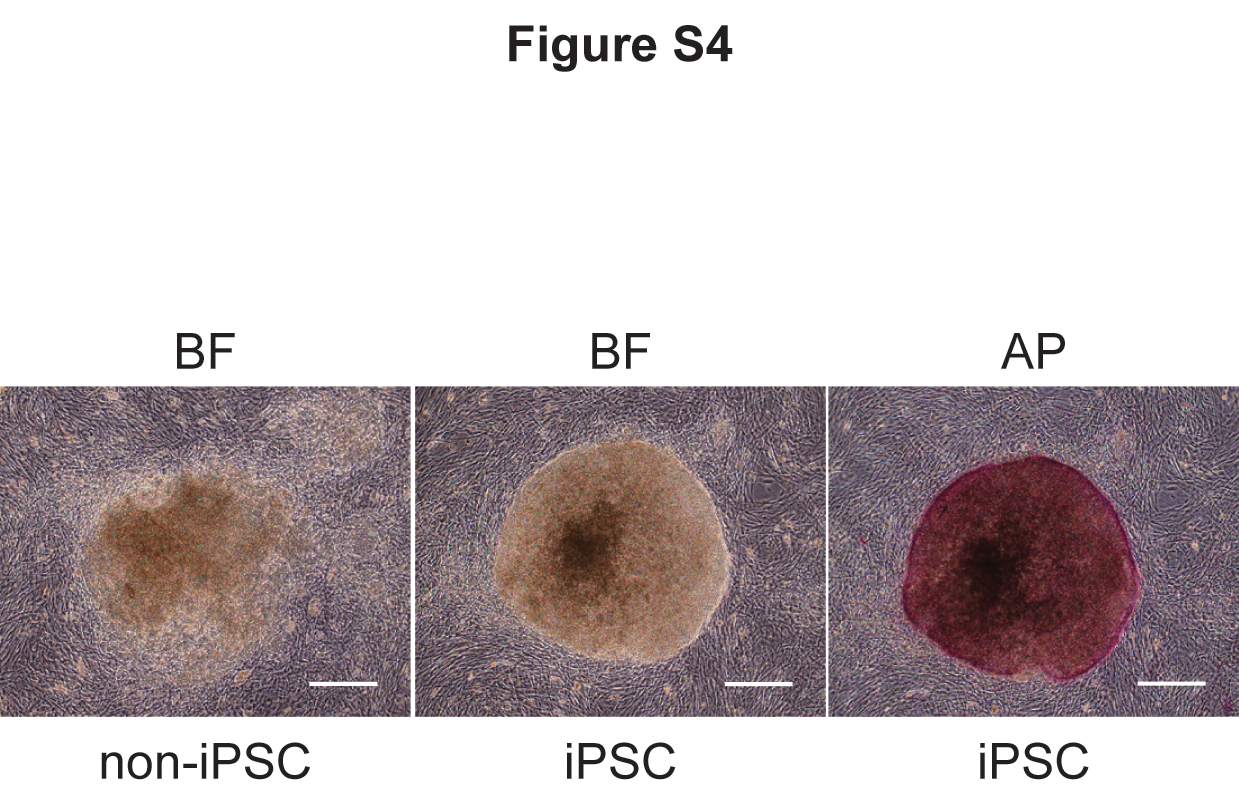

Supplement: Figure S4 — Bright-field (BF) images of an intermediate-stage colony (non-iPSC) and an iPSC colony stained positive for alkaline phosphatase. These were typical colonies observed when fibroblasts were episomally reprogrammed with small molecule-supplemented CM100 on MEF feeder cells. (TIF) [file pone.0017557.s004.tif]
