# Supplementary material for: Efficient Feeder-Free Episomal Reprogramming with Small Molecules
Source: PLoS One. 2011 Mar 1;6(3):e17557. doi: 10.1371/journal.pone.0017557 (PMC3046978; doi:10.1371/journal.pone.0017557)
Supplement: Table S1 — Primers for PCR, RT-PCR and bisulfite-sequencing PCR. (DOC) [file pone.0017557.s005.doc]

**Table S1**. Primers for PCR, RT-PCR and bisulfite-sequencing PCR.

| **Genes** | **Size (bp)** | **Symmbol** | **Sequences (5' to 3')** |  |
| --- | --- | --- | --- | --- |
| ***For quantitative RT-PCR*** | | | | |
| OCT4 | 161 | OCT4-F1 | CAGTGCCCGAAACCCACAC | Total |
|  |  | OCT4-R1 | GGAGACCCAGCAGCCTCAAA |  |
|  | 113 | OCT4-F2 | AGTTTGTGCCAGGGTTTTTG | Endogenous |
|  |  | OCT4-R2 | ACTTCACCTTCCCTCCAACC |  |
| NANOG | 111 | NANOG-F1 | CAGAAGGCCTCAGCACCTAC | Total |
|  |  | NANOG-R1 | ATTGTTCCAGGTCTGGTTGC |  |
|  | 194 | NANOG-F2 | TTTGGAAGCTGCTGGGGAAG | Endogenous |
|  |  | NANOG-R2 | GATGGGAGGAGGGGAGAGGA |  |
| SOX2 | 189 | SOX2-F2 | AGTCTCCAAGCGACGAAAAA | Endogenous |
|  |  | SOX2-R2 | TTTCACGTTTGCAACTGTCC |  |
| LIN28 | 104 | LIN28-F2 | AGTGGCCTGGATAGGGAAGT | Endogenous |
|  |  | LIN28-R2 | CTTGGCTCCATGAATCTGGT |  |
| GAPDH | 152 | GAPDH-F | GTGGACCTGACCTGCCGTCT | Endogenous |
|  |  | GAPDH-R | GGAGGAGTGGGTGTCGCTGT |  |
| ***For regular RT-PCR*** | | | | |
| T- OCT4 | 657 | Oct4-SF1 | AGTGAGAGGCAACCTGGAGA | Exogenous |
|  |  | IRES2-SR | AGGAACTGCTTCCTTCACGA |  |
| T-NANOG | 732 | Nanog-F1 | CAGAAGGCCTCAGCACCTAC | Exogenous |
|  |  | IRES2-SR | AGGAACTGCTTCCTTCACGA |  |
| T1-KLF4 | 442 | Klf4-SF1 | CCCACACAGGTGAGAAACCT | Exogenous |
|  |  | IRES2-SR | AGGAACTGCTTCCTTCACGA |  |
| T2-KLF4 | 253 | IRES2-SF | TGGCTCTCCTCAAGCGTATT | Exogenous |
|  |  | Klf4-SR | GTGGAGAAAGATGGGAGCAG |  |
| T-SV40LT | 491 | SV40T-SF1 | TGGGGAGAAGAACATGGAAG | Exogenous |
|  |  | IRES2-SR | AGGAACTGCTTCCTTCACGA |  |
| T-SOX2 | 498 | IRES2-SF | TGGCTCTCCTCAAGCGTATT | Exogenous |
|  |  | Sox2-SR | GCTTAGCCTCGTCGATGAAC |  |
| T-LIN28 | 245 | IRES2-SF | TGGCTCTCCTCAAGCGTATT | Exogenous |
|  |  | Lin28-SR | GCAAACTGCTGGTTGGACAC |  |
| T-c-MYC | 298 | IRES2-SF | TGGCTCTCCTCAAGCGTATT | Exogenous |
|  |  | Myc-SR | CACCGAGTCGTAGTCGAGGT |  |
| OCT4 | 113 | OCT4-F2 | AGTTTGTGCCAGGGTTTTTG | Endogenous |
|  |  | OCT4-R2 | ACTTCACCTTCCCTCCAACC |  |
| GAPDH | 152 | GAPDH-F | GTGGACCTGACCTGCCGTCT | Endogenous |
|  |  | GAPDH-R | GGAGGAGTGGGTGTCGCTGT |  |
| ***For PCR*** | | | | |
| T- OCT4 | 657 | Oct4-SF1 | AGTGAGAGGCAACCTGGAGA | Exogenous |
|  |  | IRES2-SR | AGGAACTGCTTCCTTCACGA |  |
| T-NANOG | 732 | Nanog-F1 | CAGAAGGCCTCAGCACCTAC | Exogenous |
|  |  | IRES2-SR | AGGAACTGCTTCCTTCACGA |  |
| T1-KLF4 | 442 | Klf4-SF1 | CCCACACAGGTGAGAAACCT | Exogenous |
|  |  | IRES2-SR | AGGAACTGCTTCCTTCACGA |  |
| T2-KLF4 | 401 | Klf4-SF1 | CCCACACAGGTGAGAAACCT | Exogenous |
|  |  | SV40pA-R | CCCCCTGAACCTGAAACATA |  |
| T-SV40LT | 491 | SV40T-SF1 | TGGGGAGAAGAACATGGAAG | Exogenous |
|  |  | IRES2-SR | AGGAACTGCTTCCTTCACGA |  |
| T-SOX2 | 534 | Sox2-SF1 | ACCAGCTCGCAGACCTACAT | Exogenous |
|  |  | SV40pA-R | CCCCCTGAACCTGAAACATA |  |
| T-LIN28 | 447 | LIN28-SF1 | AAGCGCAGATCAAAAGGAGA | Exogenous |
|  |  | SV40pA-R | CCCCCTGAACCTGAAACATA |  |
| T-c-MYC | 352 | Myc-SF1 | TCAAGAGGCGAACACACAAC | Exogenous |
|  |  | BGH-SR | CAACAGATGGCTGGCAACTA |  |
| OCT4 | 113 | Oct4-F2 | AGTTTGTGCCAGGGTTTTTG | Endogenous |
|  |  | Oct4-R2 | ACTTCACCTTCCCTCCAACC |  |
| ***For bisulfite-sequencing PCR*** | | | | |
| OCT4 | 221 | Oct4-mF3 | ATTTGTTTTTTGGGTAGTTAAAGGT | Endogenous |
|  |  | Oct4-mR3 | CCAACTATCTTCATCTTAATAACATCC |  |
| NANOG | 164 | Nanog-mF3 | TTAATTTATTGGGATTATAGGGGTG | Endogenous |
|  |  | Nanog-mR3 | AAACCTAAAAACAAACCCAACAAC |  |
|  | 295 | Nanog-mF4 | GGTTGGTTTTAAATTTTTGATTTTAG | Endogenous |
|  |  | Nanog-mR4 | ACCAATCTCACCAAAACCATTATAA |  |
