# Supplementary material for: Efficient Feeder-Free Episomal Reprogramming with Small Molecules
Source: PLoS One. 2011 Mar 1;6(3):e17557. doi: 10.1371/journal.pone.0017557 (PMC3046978; doi:10.1371/journal.pone.0017557)
Supplement: Table S2 — Antibodies for flow cytometry analysis. (DOC) [file pone.0017557.s006.doc]

**Table S2**. Antibodies for flow cytometry analysis.

| **Antigen** | **Fluorochrome** | **Clone** | **Isotype** | **Company** |
| --- | --- | --- | --- | --- |
| SSEA-3 | PE | MC631 | Rat IgM | BD Biosciences |
| SSEA-4 | PE | MC813-70 | Mouse IgG3 | BD Biosciences |
| TRA-1-60 | FITC | TRA-1-60 | Mouse IgM | BD Biosciences |
| TRA-1-81 | FITC | TRA-1-81 | Mouse IgM | BD Biosciences |
| CD44 | APC | G44-26 | Mouse IgG2b | BD Biosciences |
